# Platelet-to-hemoglobin ratio and stroke prognosis in older adults: a nonlinear and inflammation-mediated association

**DOI:** 10.3389/fmed.2025.1643860

**Published:** 2025-09-30

**Authors:** Huang Luwen, Liu Yu, Yu Ming, Xu Lei

**Affiliations:** Department of Neurology, Suining Central Hospital, Suining, Sichuan Province, China

**Keywords:** platelet-to-hemoglobin ratio, hs-CRP, unfavorable outcomes, acute ischemic stroke, nonlinear association

## Abstract

**Background:**

The platelet-to-hemoglobin ratio (PHR) has been suggested as a prognostic biomarker in several diseases, but its relevance to short-term outcomes in older patients with acute ischemic stroke (AIS) remains uncertain. This study aimed to assess the association between the PHR and 3-month unfavorable outcomes in AIS patients.

**Methods:**

We analyzed data from 1,470 older patients with AIS admitted to Seoul National University Hospital between 2010 and 2016. The primary outcome was a 3-month unfavorable outcome, defined as a modified Rankin scale score ≥3. The associations between the PHR and unfavorable outcomes were assessed using multivariable logistic regression. Receiver operating characteristic (ROC) curve analysis and bootstrap mediation analysis were also conducted.

**Results:**

A total of 462 older patients (31.43%) experienced unfavorable outcomes. A nonlinear relationship between the PHR and patient prognosis was identified. While no significant association was observed below a threshold of 1.217, the risk of unfavorable outcomes increased significantly beyond this threshold (OR = 1.479; 95% CI: 1.158, 1.888). The area under the ROC curve for the PHR was 0.59 (95% CI, 0.558, 0.622), which was greater than that of the platelet count or hemoglobin alone in predicting unfavorable outcomes. Subgroup analysis revealed that the association was stronger in patients with hyperlipidemia. Bootstrap mediation analysis further revealed that high-sensitivity C-reactive protein (hs-CRP) partially mediated the relationship between PHR and adverse outcomes.

**Conclusion:**

A nonlinear association was identified between the PHR and 3-month unfavorable outcomes in older patients with AIS. Subgroup analysis revealed that this association was more significant in patients with hyperlipidemia. Furthermore, mediation analysis indicated that hs-CRP partially mediated this relationship. These findings support the potential utility of the PHR as a practical biomarker for early prognostic stratification in AIS patients.

## 1 Introduction

Acute ischemic stroke (AIS) is a leading cause of mortality and long-term disability worldwide, particularly among older adults (1, 2). Accurate early prognostication is essential for optimizing treatment decisions and poststroke care. Although clinical tools such as the national institutes of health stroke scale (NIHSS) and the modified Rankin scale (mRS) are widely used to assess neurological deficits and outcomes (3), these assessments may be limited by timing and lack of biological insight. Moreover, a recent study revealed that the inclusion of the NIHSS score and mRS score contributes only modestly to the prediction of 30-day unplanned readmission or mortality following stroke (4). In this context, hematologic biomarkers such as the neutrophil-to-lymphocyte ratio (NLR) (5), monocyte-to-lymphocyte ratio (MLR) (6), and systemic immune-inflammation index (SII) (7) have received increasing attention because of their clinical accessibility, cost-effectiveness, and reported associations with poor outcomes in patients with AIS.

Among these, the platelet-to-hemoglobin ratio (PHR) (8, 9), which is derived from routine complete blood counts, has emerged as a novel composite indicator. Platelet (10) and hemoglobin levels (11) are independently associated with stroke outcomes, and elevated PHR has been linked to adverse outcomes in cardiovascular and systemic conditions such as myocardial infarction (8), heart failure (9), pulmonary embolism (12), and cancer (13). However, its prognostic value in AIS remains uncertain.

Therefore, this study aimed to investigate the associations between PHR and 3-month functional outcomes in a large cohort of older patients with AIS. In addition, we investigated whether high-sensitivity C-reactive protein (hs-CRP), a well-established marker of systemic inflammation (14), mediates this association, with the aim of elucidating potential biological pathways linking the PHR to stroke prognosis.

## 2 Methods

### 2.1 Study design and population

**Figure 1:**
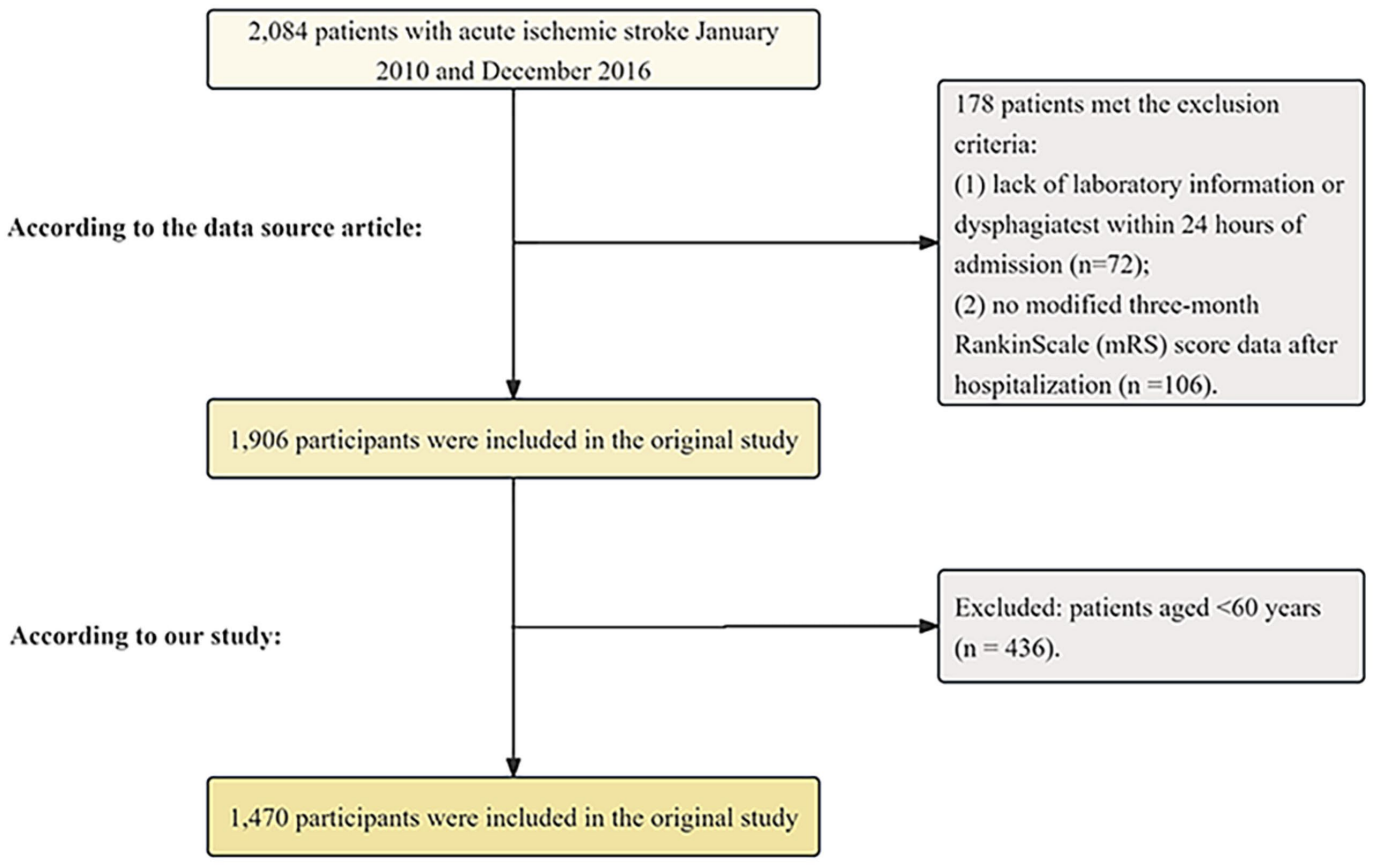
Flow chart of the study population.

The study analyzed data from a prospective registry system at Seoul National University Hospital, collected between January 2010 and December 2016 (15). Initially, 2,084 patients with AIS admitted within 7 days of symptom onset were identified through a prospective registry. The exclusion criteria included missing laboratory or dysphagia screening data within 24 h of admission (*n* = 72) and the absence of three-month mRS scores after discharge (*n* = 106). Patients younger than 60 years (*n* = 436) were also excluded. After applying these criteria, a total of 1,470 patients were included in the final analysis (Figure 1).

This study was approved by the Institutional Review Board of Seoul National University Hospital (IRB No. 1009-062-332), and the requirement for informed consent was waived (15).

### 2.2 Exposure and outcome definitions

The exposure variable, PHR, was calculated using the following formula: PHR = platelet (10^9^/L)/hemoglobin (g/L) (8).

For example, for a patient with a platelet count of 219 × 10^9^/L and a hemoglobin concentration of 132.5 g/L, the PHR would be calculated as follows:

PHR = 219/132.5 = 1.653. Thus, the PHR for this patient would be 1.653.

The primary outcome was an unfavorable outcome at 3 months, defined as an mRS score of 3 or higher (16). Relevant data were retrieved from outpatient records, and structured telephone interviews were conducted 3 months post-AIS (15).

### 2.3 Covariate assessments

Covariates included demographic and lifestyle factors (gender, age, body mass index [BMI], and smoking status); comorbidities (hypertension, diabetes mellitus [DM], prior stroke or transient ischemic attack [TIA], coronary heart disease [CHD], hyperlipidemia, and atrial fibrillation [AF]); clinical characteristics (stroke etiology, admission mRS score, and admission NIHSS score); and laboratory parameters such as white blood cell count (WBC), hemoglobin (HGB), hematocrit (HCT), fibrinogen (FIB), platelet count (PLT), mean corpuscular volume (MCV), triglycerides (TG), total cholesterol (TC), high-density lipoprotein cholesterol (HDL-C), low-density lipoprotein cholesterol (LDL-C), blood urea nitrogen, serum creatinine (Scr), alanine aminotransferase (ALT), fasting blood glucose (FBG), and hs-CRP. The parameter settings for each indicator are detailed in Supplementary Table S1.

Since the study population comprised individuals aged >60 years with no missing data for the relevant variables, complete-case analysis was applied.

### 2.4 Statistical analysis

Baseline characteristics are summarized as the means ± standard deviations (SDs) or medians with interquartile ranges (IQRs) for continuous variables and as frequencies with percentages for categorical variables. For continuous variables, Student’s t test or one-way ANOVA was applied when normally distributed, and the Kruskal–Wallis test was used for nonnormally distributed data. Categorical variables were compared via the chi-square test or Fisher’s exact test, as appropriate.

To evaluate potential collinearity between the PHR and other covariates, we calculated the generalized variance inflation factor (GVIF) and adjusted GVIF (Supplementary Table S2). The results indicated no evidence of significant collinearity. Logistic regression models were used to investigate the associations between the PHR and adverse outcomes. Three models were constructed: Model 1 was unadjusted; Model 2 was adjusted for age and gender; and Model 3 was further adjusted for BMI, WBC, ALT, BUN, Scr, LDL, FBG, smoking, previous stroke/TIA, hypertension, DM, hyperlipidemia, AF, CHD, stroke etiology, and the NIHSS score at admission. A restricted cubic spline (RCS) regression was subsequently conducted on the basis of the fully adjusted model to assess the potential dose–response relationship between the PHR and adverse outcomes. To evaluate and compare the predictive performance of the PHR, HGB, and PLT levels, receiver operating characteristic (ROC) curves were generated. The area under the ROC curve (AUC) was used to quantify the discriminative ability of each variable. Two models were compared to assess the added value of PHR over standard clinical tools. Model 1 included the following variables: age, sex, NIHSS, hypertension, diabetes, and coronary artery disease. In Model 2, PHR was incorporated as an additional predictor. The performance improvement was further evaluated using Net Reclassification Improvement (NRI) and Integrated Discrimination Improvement (IDI). A LRT was also performed to compare the two models. Additionally, a bootstrap-corrected AUC (≥1,000 resamples) was calculated for each model to assess the stability of the AUC values and evaluate the model performance under resampling conditions. To assess the clinical applicability of the models, a calibration analysis was conducted.

To further assess the robustness of our findings, we conducted sensitivity analyses, including subgroup analyses stratified by gender, age, BMI, smoking status, hypertension, DM, CHD, hyperlipidemia, AF, and NIHSS score at admission, to evaluate whether the associations between the PHR and adverse outcomes in patients with AIS remained consistent across different clinical subgroups. In addition, E values were calculated to estimate the minimum strength of association that an unmeasured confounder would need to have with both PHR and adverse outcomes to fully explain the observed associations via the formula E = RR + sqrt[RR × (RR − 1)] (17).

Mediation analysis was conducted to assess whether high-sensitivity C-reactive protein (hs-CRP) mediated the relationship between the platelet-to-hemoglobin ratio (PHR) and unfavorable 3-month outcomes. A regression-based approach with 5,000 bootstrap resamples was used to estimate direct, indirect, and total effects. Statistical significance was determined using 95% bias-corrected confidence intervals, and all the models were adjusted for relevant confounders. The proportion of the total effect mediated by hs-CRP was also calculated.

All the statistical analyses were performed via R software (version 4.2.2) and Free Statistics software (version 2.1). A two-sided *p* < 0.05 was considered statistically significant.

## 3 Results

### 3.1 Baseline characteristics

In this study, 1,470 older patients with AIS were enrolled and categorized into tertiles on the basis of PHR levels. With increasing PHR, the proportions of female patients and those with DM significantly increased, whereas the prevalence of smoking and AF decreased. The distribution of stroke subtypes also differed significantly across PHR tertiles (all *p* < 0.001) (Table 1). Among the 462 patients with unfavorable outcomes at 3 months (*n* = 462), female, age ≥ 80 years, BMI > 30 kg/m^2^, smoking history, hypertension, DM, previous stroke or TIA, AF, and higher baseline scores on both the mRS and the NIHSS were all significantly more prevalent (all *p* < 0.05). Laboratory parameters, including WBC, fibrinogen, and hs-CRP, were significantly elevated in the unfavorable outcome group (all *p* < 0.001), and the PHR was also significantly greater than that in the favorable outcome group (1.78 ± 0.81 vs. 1.66 ± 0.57, *p* = 0.001) (Supplementary Table S3). Univariate logistic regression analysis demonstrated that female sex, age ≥80 years, DM, prior stroke or TIA, AF, higher NIHSS scores, and elevated levels of fibrinogen and hs-CRP were significantly associated with an increased risk of unfavorable outcomes (Supplementary Table S4).

**Table 1:** Patient demographics and baseline characteristics.

Characteristic	Total	PHR quartiles			*p*
		Q1	Q2	Q3	
Participants	1,470	500	489	481	
Gender					<0.001
Male	854 (58.10)	362 (72.4)	289 (59.1)	203 (42.2)	
Female	616 (41.90)	138 (27.6)	200 (40.9)	278 (57.8)	
Age (years)					0.271
60 to <70	505 (34.35)	169 (33.8)	175 (35.79)	161 (33.47)	
70 to <80	670 (45.58)	238 (47.6)	224 (45.81)	208 (43.24)	
≥80	295 (20.07)	93 (18.6)	90 (18.4)	112 (23.28)	
BMI (kg/m^2^)					0.053
<25	1,080 (73.47)	348 (69.6)	360 (73.62)	372 (77.34)	
25–29.9	358 (24.35)	141 (28.2)	115 (23.52)	102 (21.21)	
>30	32 (2.18)	11 (2.2)	14 (2.86)	7 (1.46)	
Smoking, *n* (%)					<0.001
No	957 (65.10)	298 (59.6)	314 (64.21)	345 (71.73)	
Yes	513 (34.90)	202 (40.4)	175 (35.79)	136 (28.27)	
Hypertension, *n* (%)					0.486
No	464 (31.56)	161 (32.2)	161 (32.92)	142 (29.52)	
Yes	1,006 (68.44)	339 (67.8)	328 (67.08)	339 (70.48)	
DM, *n* (%)					0.008
No	966 (65.71)	337 (67.4)	339 (69.33)	290 (60.29)	
Yes	504 (34.29)	163 (32.6)	150 (30.67)	191 (39.71)	
Previous stroke/TIA, *n* (%)					0.24
No	1,125 (76.53)	372 (74.4)	386 (78.94)	367 (76.3)	
Yes	345 (23.47)	128 (25.6)	103 (21.06)	114 (23.7)	
CHD, *n* (%)					0.523
No	1,275 (86.73)	429 (85.8)	422 (86.3)	424 (88.15)	
Yes	195 (13.27)	71 (14.2)	67 (13.7)	57 (11.85)	
Hyperlipidemia, *n* (%)					0.406
No	948 (64.49)	334 (66.8)	311 (63.6)	303 (62.99)	
Yes	522 (35.51)	166 (33.2)	178 (36.4)	178 (37.01)	
Atrial fibrillation, *n* (%)					<0.001
No	1,099 (74.76)	335 (67)	376 (76.89)	388 (80.67)	
Yes	371 (25.24)	165 (33)	113 (23.11)	93 (19.33)	
Stroke etiology, *n* (%)					<0.001
LAA	477 (32.45)	132 (26.4)	168 (34.36)	177 (36.8)	
SVO	288 (19.59)	91 (18.2)	112 (22.9)	85 (17.67)	
CE	427 (29.05)	196 (39.2)	121 (24.74)	110 (22.87)	
Other determined	98 (6.67)	27 (5.4)	27 (5.52)	44 (9.15)	
Undetermined	180 (12.24)	54 (10.8)	61 (12.47)	65 (13.51)	
mRS at admission, *n* (%)					0.401
0	1,058 (72.02)	353 (70.74)	371 (75.87)	334 (69.44)	
1	130 (8.85)	45 (9.02)	45 (9.2)	40 (8.32)	
2	90 (6.13)	31 (6.21)	24 (4.91)	35 (7.28)	
3	80 (5.45)	31 (6.21)	20 (4.09)	29 (6.03)	
4	61 (4.15)	19 (3.81)	17 (3.48)	25 (5.2)	
5	50 (3.40)	20 (4.01)	12 (2.45)	18 (3.74)	
mRS at admission, *n* (%)					0.436
≤2	220 (53.53)	76 (52.05)	69 (58.47)	75 (51.02)	
≥3	191 (46.47)	70 (47.95)	49 (41.53)	72 (48.98)	
NIHSS score at admission, *n* (%)					0.013
≤5	864 (58.78)	279 (55.8)	310 (63.39)	275 (57.17)	
5 to ≤13	469 (31.90)	159 (31.8)	145 (29.65)	165 (34.3)	
>13	137 (9.32)	62 (12.4)	34 (6.95)	41 (8.52)	
Laboratory parameters					
WBC (10^9/L)	8.04 ± 2.88	7.60 ± 2.79	7.76 ± 2.62	8.77 ± 3.07	<0.001
HGB (g/L)	132.5 ± 19.6	139.2 ± 19.4	135.9 ± 15.8	122.5 ± 19.4	<0.001
HCT (%)	39.49 ± 5.52	41.24 ± 5.42	40.40 ± 4.53	36.76 ± 5.51	<0.001
FIB (mg/L)	332.93 ± 92.03	316.25 ± 81.46	323.32 ± 79.01	360.03 ± 107.49	<0.001
PLT (×10^9^/L)	219.04 ± 69.99	158.58 ± 35.60	214.58 ± 28.02	286.42 ± 67.07	<0.001
MCV	93.34 ± 5.03	93.95 ± 5.12	93.68 ± 4.52	92.37 ± 5.30	<0.001
TG (mg/dl)	101.68 ± 55.26	96.71 ± 55.86	105.23 ± 54.66	103.29 ± 54.97	0.039
TC (mg/dl)	175.59 ± 42.77	167.82 ± 42.38	180.57 ± 38.60	178.62 ± 46.05	<0.001
HDL-C (mg/dl)	43.98 ± 16.67	43.10 ± 16.80	45.38 ± 16.13	43.47 ± 17.03	0.071
LDL-C (mg/dl)	101.68 ± 41.35	96.33 ± 40.70	104.43 ± 38.01	104.46 ± 44.68	0.002
BUN (mg/dl)	18.43 ± 9.34	18.79 ± 8.92	17.74 ± 8.37	18.74 ± 10.60	0.139
Scr (mg/dl)	0.90 (0.74, 1.11)	0.96 (0.81, 1.13)	0.89 (0.74, 1.10)	0.84 (0.70, 1.09)	<0.001
ALT (U/L)	18.00 (13.00, 25.00)	20.00 (14.00, 28.25)	18.00 (13.00, 24.00)	16.00 (11.00, 23.00)	<0.001
FBG (mg/dl)	99.17 ± 45.86	99.87 ± 45.76	100.86 ± 44.53	96.72 ± 47.25	0.342
hs-CRP (mg/L)	0.13 (0.04, 0.48)	0.12 (0.04, 0.46)	0.09 (0.03, 0.32)	0.20 (0.06, 0.92)	<0.001
Outcomes					0.002
Unfavorable outcomes, n (%)	1,008 (68.57)	347 (69.4)	358 (73.21)	303 (62.99)	
Favorable outcomes, n (%)	462 (31.43)	153 (30.6)	131 (26.79)	178 (37.01)	
PHR	1.69 ± 0.66	1.14 ± 0.20	1.58 ± 0.11	2.39 ± 0.67	<0.001

Variables are presented as the means ± SDs, medians (IQRs) or *n* (%). BMI, body mass index; DM, diabetes mellitus; TIA, transient ischemic attack; CHD, coronary heart disease; LAA, large-artery atherosclerosis; SVO, small vessel occlusion; CE, cardioembolism; mRS, modified Rankin scale; NIHSS, national institutes of health stroke scale; WBC, white blood cell; HGB, hemoglobin; HCT, hematocrit; FIB, fibrinogen; PLT, platelet; MCV, mean corpuscular volume; TG, triglyceride; TC, total cholesterol; HDL-C, high-density lipoprotein cholesterol; LDL-C, low-density lipoprotein cholesterol; BUN, blood urea nitrogen; Scr, serum creatinine; ALT, alanine aminotransferase; FBG, fasting blood glucose; hs-CRP, high-sensitivity C-reactive protein; PHR, platelet-to-hemoglobin ratio.

### 3.2 Association between the PHR and unfavorable outcomes

**Figure 2:**
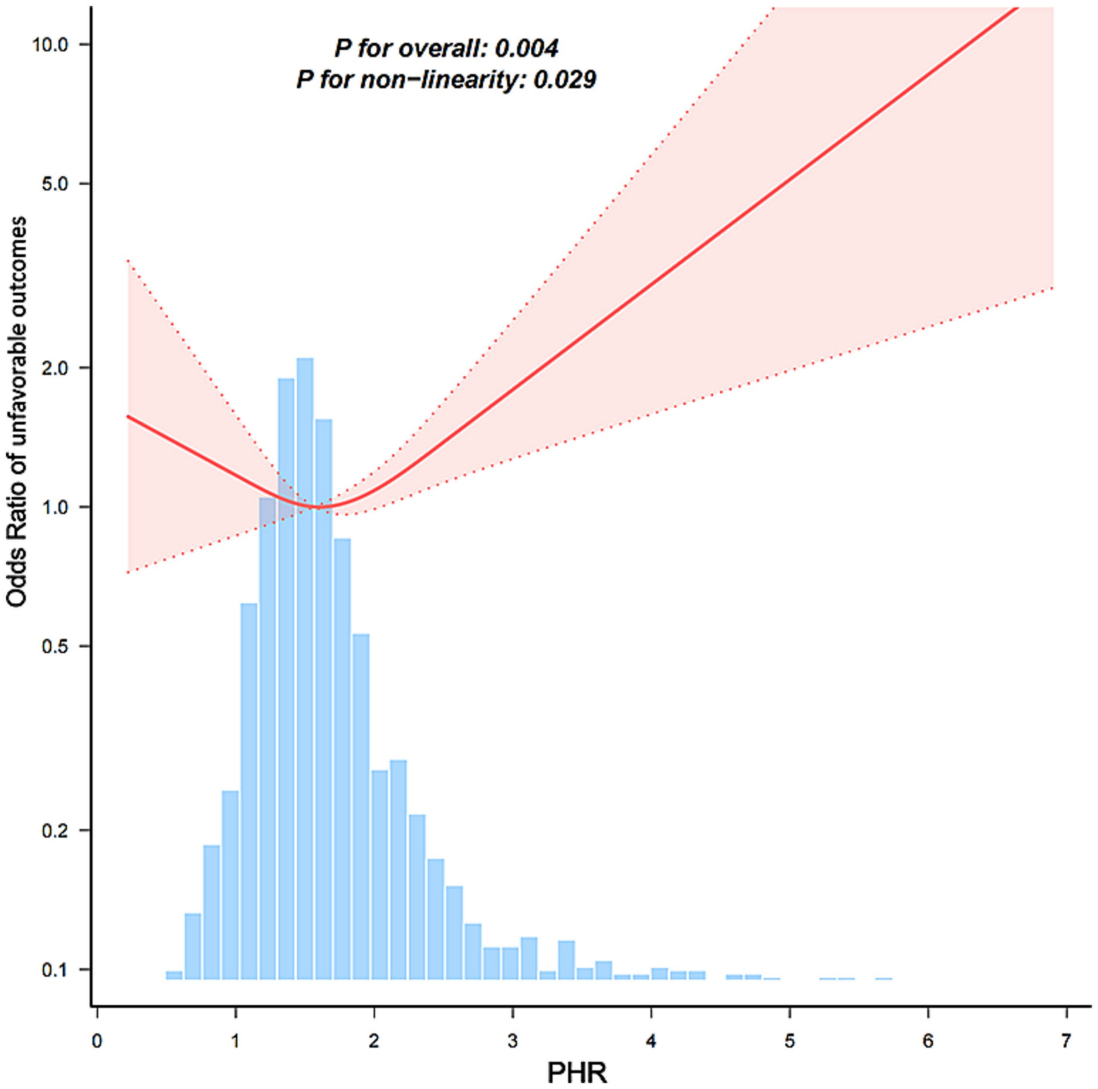
Association between the PHR and unfavorable outcomes 3 months after AIS. The model was adjusted for age, gender, BMI, WBC, ALT, BUN, Scr, LDL-C, FBG, smoking, previous stroke/TIA, hypertension, DM, hyperlipidemia, AF, CHD, stroke etiology, and NIHSS score at admission.

Multivariate logistic regression analysis revealed that a higher PHR was significantly associated with an increased risk of unfavorable outcomes at 3 months after AIS (Table 2). According to the fully adjusted model, each unit increase in the PHR was associated with a 30.9% greater risk (OR = 1.309, 95% CI: 1.067, 1.607). Patients in Q3 had a significantly greater risk than those in Q1 did (OR = 1.428, 95% CI: 1.021, 1.999), with a significant linear trend across tertiles (*p* for trend = 0.0374). RCS analysis further revealed a nonlinear association between the PHR and the risk of unfavorable outcomes, with an inflection point observed at approximately 1.217 (*p* for nonlinear = 0.043) (Figure 2). Below this point, the association was inverse but not statistically significant (OR = 0.257, 95% CI: 0.037, 1.790), whereas above the inflection point, the risk increased significantly with increasing PHR (OR = 1.479, 95% CI: 1.158, 1.888) (Table 3).

**Table 2:** Association between PHR and unfavorable outcomes 3 months after stroke in different models.

Characteristic	Event, (*n* %)	Model 1		Model 2		Model 3	
		OR (95%CI)	*p*	OR (95%CI)	*p*	OR (95%CI)	*p*
PHR (per 1 unit)	462 (31.4)	1.303 (1.107, 1.535)	0.002	1.237 (1.044, 1.466)	0.014	1.309 (1.067, 1.607)	0.01
PHR							
Q1 (<1.39)	153 (30.6)	1 (Ref)		1 (Ref)		1 (Ref)	
Q2 (1.39–1.78)	131 (26.8)	0.83 (0.63, 1.094)	0.186	0.79 (0.594, 1.051)	0.105	0.996 (0.716, 1.384)	0.979
Q3 (>1.78)	178 (37)	1.332 (1.022, 1.737)	0.034	1.16 (0.876, 1.537)	0.3	1.428 (1.021, 1.999)	0.038
*p* for trend	462 (31.4)	1.158 (1.012, 1.326)	0.033	1.08 (0.936, 1.245)	0.293	1.197 (1.011, 1.417)	0.037

Model 1: We did not adjust for other covariates; Model 2: Age, and gender; Model 3: Age, gender, BMI, WBC, ALT, BUN, Scr, LDL-C, FBG, smoking, previous stroke/TIA, hypertension, DM, hyperlipidemia, AF, CHD, stroke etiology, and NIHSS score at admission. BMI, body mass index; WBC, white blood cell; ALT, alanine aminotransferase; BUN, blood urea nitrogen; Scr, serum creatinine; LDL-C, low-density lipoprotein cholesterol; FBG, fasting blood glucose; TIA, transient ischemic attack; DM, diabetes mellitus; AF, atrial fibrillation; CHD, coronary heart disease; NIHSS, national institutes of health stroke scale.

**Table 3:** Threshold-effect analysis of the relationship between the baseline PHR level and unfavorable outcomes 3 months after AIS.

Models	Per-unit increase		
	OR	95%CI	*p*
Model I	1.309	1.067, 1.607	0.01
One line effect			
Model II			
Turning point (K)	1.217	1.01, 1.425	
Baseline PHR levels < K	0.257	0.037, 1.79	0.170
Baseline PHR levels > K	1.479	1.158, 1.888	0.002
*p* value for LRT test∗			0.043

Model I, one-line linear regression model; Model II, two-piece linear regression model. Adjusted for age, gender, BMI, WBC, ALT, BUN, Scr, LDL-C, FBG, smoking, previous stroke/TIA, hypertension, DM, hyperlipidemia, AF, CHD, stroke etiology, and NIHSS score at admission. ∗*p* < 0.05 indicates that Model II is significantly different from Model I. LRT, logarithm likelihood ratio test; BMI, body mass index; WBC, white blood cell; ALT, alanine aminotransferase; BUN, blood urea nitrogen; Scr, serum creatinine; LDL-C, low-density lipoprotein cholesterol; FBG, fasting blood glucose; TIA, transient ischemic attack; DM, diabetes mellitus; AF, atrial fibrillation; CHD, coronary heart disease; NIHSS, national institutes of health stroke scale.

**Figure 3:**
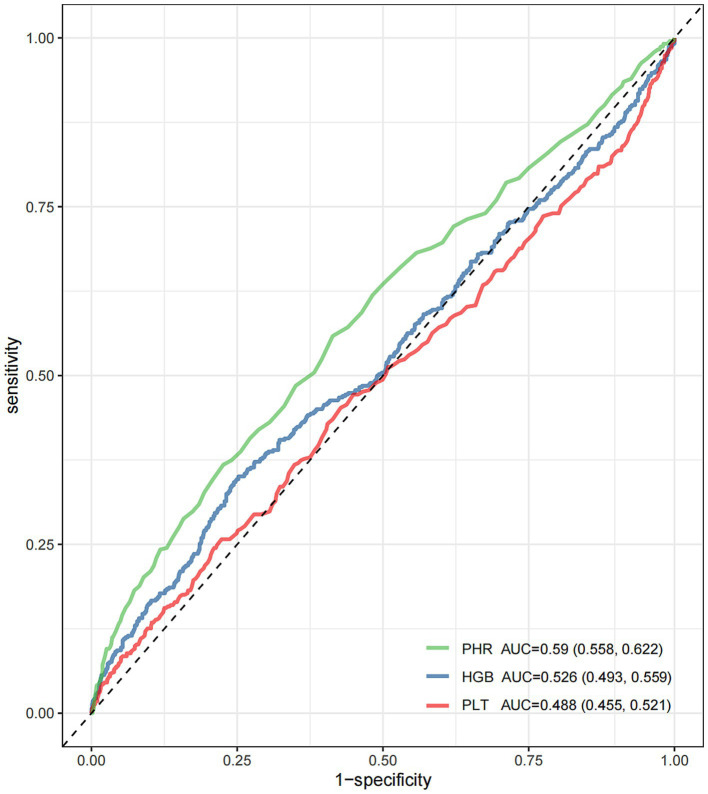
ROC curves comparing the predictive efficacy of the PHR, HGB, and PLT for unfavorable outcomes. PHR, platelet-to-hemoglobin ratio; HGB, hemoglobin; PLT, platelet.

**Figure 4:**
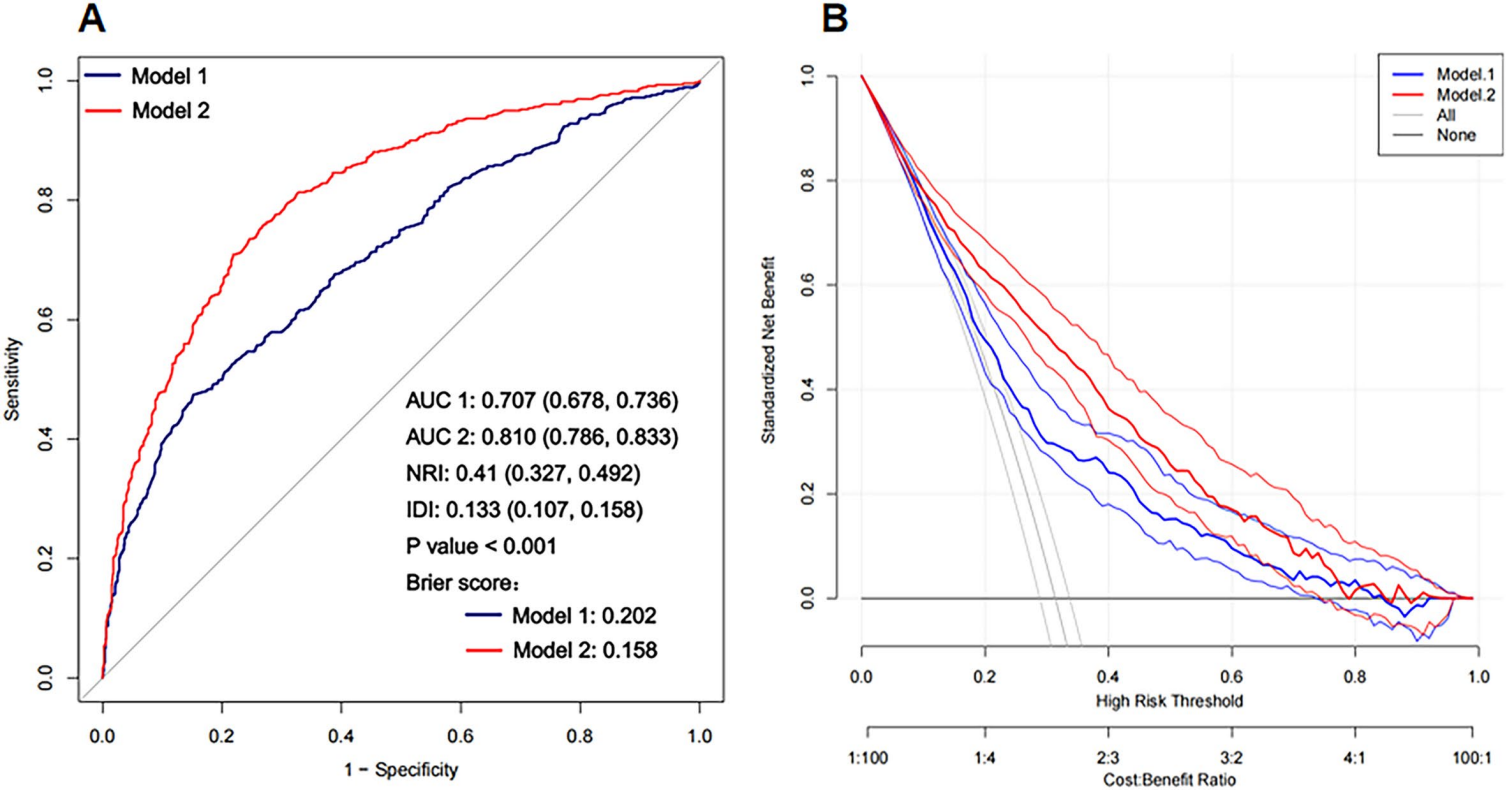
ROC curve analysis and improvement index comparison of Model 1 and Model 2 in predicting adverse outcomes. **(A)** Displays the receiver operating characteristic (ROC) curves for Model 1 and Model 2; **(B)** Analyzes the performance improvement of Model 1 and Model 2 in logistic regression.

ROC analysis revealed that among the three biomarkers evaluated, the PHR had the greatest ability to predict unfavorable outcomes at 3 months (Figure 3 and Supplementary Table S5). The PHR demonstrated the highest area under the curve (AUC = 0.59, 95% CI: 0.558, 0.622), followed by HGB (AUC = 0.526, 95% CI: 0.493, 0.559), whereas the PLT had the lowest predictive performance (AUC = 0.488, 95% CI: 0.455, 0.521). We observed the following AUC values for the two models: Model 1: AUC = 0.707 (95% CI, 0.678, 0.736) and Model 2: AUC = 0.810 (95% CI, 0.786, 0.833). While the inflection point (PHR ≈ 1.217) identified by the RCS analysis indicates a trend in the relationship between BUCR and mortality risk, the optimal threshold derived from the ROC curve (PHR ≈ 1.869) serves as a clear cut-off for classifying patients into high-risk and low-risk categories, providing a distinct risk stratification threshold. To further assess the improvements in model performance, we calculated NRI = 0.41 (95% CI, 0.327, 0.492) and IDI = 0.133 (95% CI, 0.107, 0.158). Additionally, a LRT was conducted to compare the two models, revealing a statistically significant improvement (*p* < 0.001) (Figure 4A). The calibration analysis further supports this finding, with the calibration curve of Model 2 closely approaching with the diagonal, demonstrating a slope near 1 and an intercept near 0 (Figure 4B). This indicates that Model 2 is more accurate in its predictions, particularly in identifying high-risk patients. Compared to the “null model” (black curve) and the “overall sample model” (gray curve), Model 2 performs better in terms of both standardized net benefit and calibration, highlighting its greater clinical applicability.

### 3.3 Subgroup analysis

**Figure 5:**
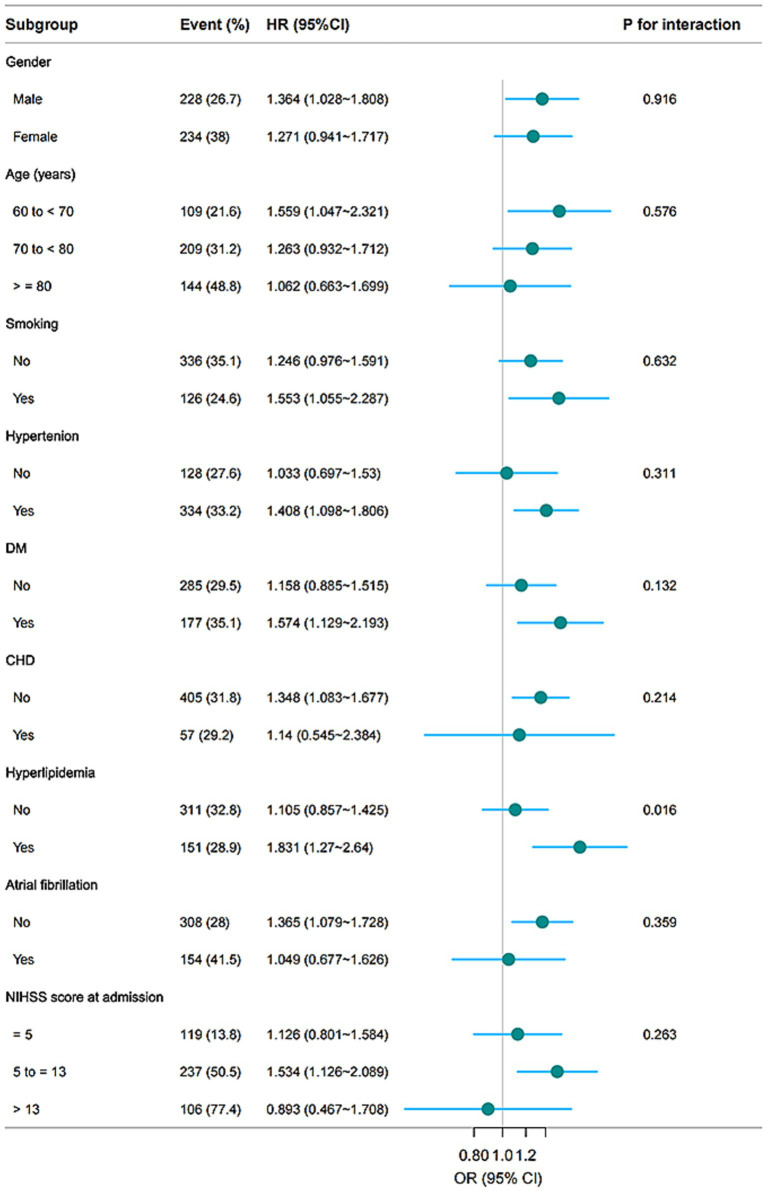
Subgroup analyses of the associations between the PHR and unfavorable outcomes 3 months after AIS. The model was adjusted for age, gender, BMI, WBC, ALT, BUN, Scr, LDL-C, FBG, smoking, previous stroke/TIA, hypertension, DM, hyperlipidemia, AF, CHD, stroke etiology, and NIHSS score at admission.

Subgroup analysis revealed that the association between elevated PHR and three-month unfavorable outcomes was consistent across most stratified subpopulations (*p* for interaction >0.05), except for hyperlipidemia (*p* for interaction = 0.016) (Figure 5). In patients with hyperlipidemia, the association was stronger (OR = 1.831, 95% CI: 1.270, 2.640), whereas no significant association was detected in those without hyperlipidemia (OR = 1.105, 95% CI: 0.857, 1.425).

### 3.4 Sensitivity analysis

The E value for PHR, calculated from Model 3, was 2.68 (Supplementary Figure S1). The bias plot indicates that an unmeasured confounder would need to be associated with both PHR and unfavorable outcomes, with a risk ratio of at least 2.21 to fully explain the observed association (RR = 1.17). These results suggest that the observed association is moderately robust to potential unmeasured confounding.

### 3.5 Mediation analysis

**Figure 6:**
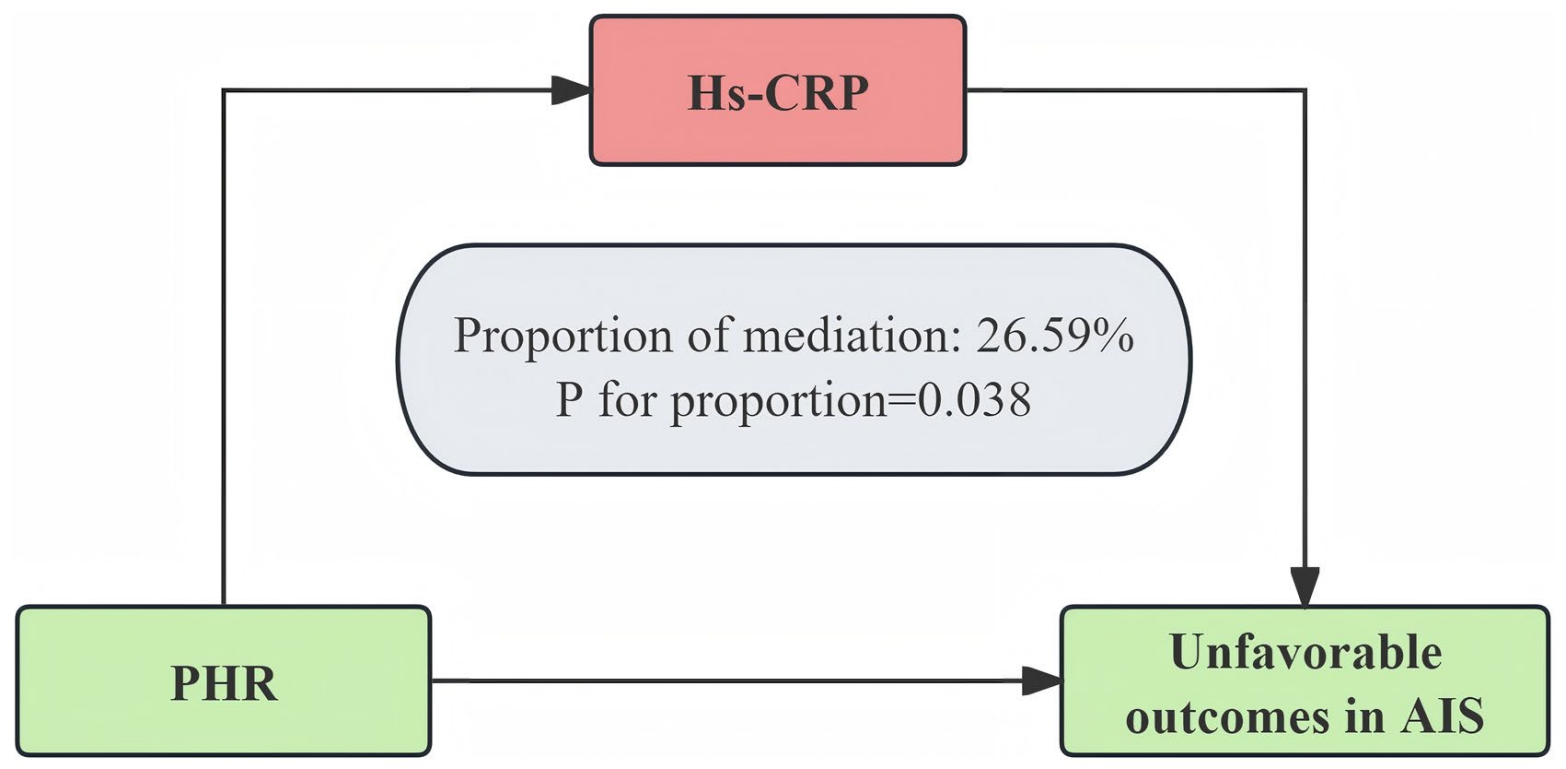
hs-CRP as a mediator of the relationship between the PHR and unfavorable outcomes 3 months after AIS. The model was adjusted for age, gender, BMI, WBC, ALT, BUN, Scr, LDL-C, FBG, smoking, previous stroke/TIA, hypertension, DM, hyperlipidemia, AF, CHD, stroke etiology, and NIHSS score at admission.

Mediation analysis indicated that the relationship between the PHR and three-month unfavorable outcomes was partially mediated by hs-CRP (Figure 6; Supplementary Table S6). The direct effect of the PHR remained statistically significant after adjusting for the mediator (95% CI, 0.0018–0.0527, *p* = 0.042), and the total effect was also significant (*p* = 0.016). Approximately 26.59% of the total effect was mediated through hs-CRP. These results were confirmed via bootstrap analysis, indicating the robustness of the mediation effect.

## 4 Discussion

In this large-scale cohort study of older patients with AIS, we identified a significant inverse association between the PHR and 3-month unfavorable outcomes. A nonlinear relationship was observed between the PHR and poor prognosis. Furthermore, mediation analysis revealed that hs-CRP partially mediated the association between the PHR and functional outcome, suggesting that an inflammatory component underlies this relationship. These results indicate that systemic inflammation and dysregulation of hematologic components may jointly contribute to stroke prognosis, suggesting potential targets for intervention.

The platelet-to-hemoglobin ratio (PHR) is a simple and cost-effective biomarker derived from routine blood tests. It combines the platelet count, which reflects prothrombotic and inflammatory activity, with the hemoglobin concentration, which indicates oxygen-carrying capacity and nutritional status. PHR has been reported to be significantly associated with adverse clinical outcomes in various diseases (8, 9, 12). For example, Ozbeyaz et al. identified the PHR as an independent predictor of in-hospital mortality in patients with acute pulmonary embolism (12). It has also demonstrated prognostic value in conditions such as ST-segment elevation myocardial infarction (8), congestive heart failure (9), rheumatoid arthritis (18), and colorectal cancer (13), suggesting broad applicability across disease types. Moreover, its individual components—platelet count and hemoglobin level—have been independently linked to stroke severity and prognosis. A 2024 study revealed a U-shaped relationship between platelet count and mortality in hemorrhagic stroke patients (10), and Kanazawa et al. reported that the baseline platelet count predicted early neurological deterioration in IS patients (19). However, another study of 281 first-ever IS patients indicated that while an elevated PLT increased stroke risk, it was not significantly associated with poststroke functional outcomes (20). In terms of hemoglobin, a large cohort study involving 14,159 patients with AIS or TIA revealed that both anemia and elevated hemoglobin levels were positively associated with mortality and poor functional outcomes (11). Furthermore, a two-sample Mendelian randomization study using UK Biobank data suggested that anemia may be a risk factor for stroke (21). Although the platelet count and hemoglobin level have been shown to individually influence stroke prognosis, research directly examining the prognostic value of the PHR in AIS patients remains limited.

On the basis of data from Seoul National University Hospital, this study investigated the association between the PHR and 3-month unfavorable outcomes in older patients with AIS. We identified a significant positive nonlinear relationship, with an inflection point at approximately 1.217. When the PHR exceeded this threshold, each one-unit increase was associated with a 47.9% greater risk of poor functional outcome, even after full adjustment for confounders. However, no significant associations were observed below this threshold. This dose–response pattern is consistent with previous findings in heart failure patients (9). The observed relationship likely reflects complex interactions among thrombogenicity, oxygen-carrying capacity, and systemic inflammation within the context of AIS. Inflammation is a well-recognized feature of AIS (22), and elevated levels of proinflammatory mediators can stimulate megakaryocyte proliferation and platelet production (23–25), indicating a heightened prothrombotic state (26). Furlan et al. found that increased platelet count in AIS patients was significantly associated with 30-day and 90-day mortality (27). A study by Yang et al., which analyzed 16,842 patients with ischemic stroke or transient ischemic attack, found that higher platelet counts were associated with an increased risk of recurrent stroke, all-cause mortality, and functional impairment within 1 year (28). Concurrently, low hemoglobin levels may exacerbate cerebral hypoxia and tissue damage following AIS, thereby affecting neuronal survival and functional recovery (29). Kimberly et al. observed that lower hemoglobin levels in AIS patients were significantly correlated with larger infarct volumes and infarct expansion (30). Other studies have also shown that low hemoglobin levels are significantly associated with increased mortality, functional disability, and recurrent stroke risk following AIS (11). Furthermore, it has been suggested that the recovery of hemoglobin levels may help reduce hospitalization time and improve functional recovery in stroke patients (31). Moreover, anemia is often accompanied by systemic inflammation, with elevated markers such as fibrinogen and von Willebrand factor enhancing platelet reactivity (32, 33). Compensatory bone marrow hyperactivity in response to anemia may also contribute to increased platelet counts (34). Collectively, these findings suggest that an elevated PHR may reflect an adverse pathophysiologic state and serve as a potential prognostic biomarker for risk stratification in older patients with AIS. In support of this finding, ROC analysis demonstrated that the PHR had a greater AUC for predicting 3-month unfavorable outcomes than the platelet count or hemoglobin alone did, indicating superior discriminative ability. As an integrated hematologic index, the PHR may therefore provide more robust prognostic insight than its individual components. Although traditional clinical tools such as NIHSS and mRS are crucial for assessing stroke severity, they primarily rely on clinical symptoms and may not fully capture the underlying biological processes that influence stroke prognosis. The PHR, a simple and cost-effective biomarker, provides additional biological insights into factors such as thrombosis, oxygen-carrying capacity, and inflammation, all of which play important roles in stroke prognosis. By integrating PLT and HGB levels, PHR serves as a complementary tool that offers early risk stratification information beyond what traditional tools alone can provide. Incorporating PHR into clinical decision-making allows healthcare providers to perform early risk stratification and implement timely interventions for high-risk patients, potentially improving patient outcomes.

Subgroup analysis revealed a significantly stronger association between the PHR and poor outcomes in patients with hyperlipidemia, suggesting that the PHR may have greater prognostic value in this population. Hyperlipidemia is known to aggravate ischemic injury through mechanisms such as endothelial dysfunction (35), heightened systemic inflammation (36), and a prothrombotic state (37), which may in turn amplify the prognostic relevance of the PHR. As a composite index incorporating the platelet count and hemoglobin level, the PHR may reflect the combined effects of inflammation, thrombogenicity, and anemia—factors that are implicated in stroke progression and outcome. Therefore, in AIS patients with concomitant hyperlipidemia, the PHR may serve as a more informative and clinically meaningful prognostic biomarker. Additionally, our mediation analysis revealed that hs-CRP significantly mediates the association between the PHR and AIS, suggesting that systemic inflammation is a key pathway linking the PHR to stroke outcomes. Elevated hs-CRP, a well-established marker of inflammation and vascular risk, has been linked to increased mortality, recurrence, and poor prognosis in AIS patients (38). These findings underscore the central role of inflammation in AIS pathophysiology and support the prognostic relevance of the PHR, which integrates both platelet activity and hemoglobin levels—components potentially modulated by inflammatory states. Our findings provide novel insights into the relationship between hematologic markers and AIS outcomes, warranting further studies to elucidate the underlying biological mechanisms.

This study is the first to demonstrate a significant nonlinear association between the PHR and 3-month functional outcomes in older patients with AIS. Notably, this relationship was more apparent in individuals with hyperlipidemia. Furthermore, mediation analysis indicated that hs-CRP partially mediated the effect of the PHR on clinical outcomes, suggesting that systemic inflammation may be an intermediate pathway. Given its simplicity, accessibility, and low cost, the PHR holds potential as a clinically applicable prognostic marker in AIS. However, several limitations of this study should be acknowledged. First, although the mRS is a widely accepted tool for assessing post-stroke functional outcomes, reliance on a single outcome measure may limit the assessment of comprehensive neurological recovery. Second, important treatment-related variables—such as intravenous thrombolysis and endovascular therapy—that may affect outcomes were not included in the present analysis. Future studies should incorporate these therapeutic factors to reduce residual confounding. Third, this study utilized the 3-month mRS as the primary endpoint, as it is a critical time point for assessing functional recovery in AIS. However, the 3-month mRS may not fully reflect long-term outcomes, as patients may experience continued recovery or deterioration beyond this period. Due to the absence of 6–12 month follow-up data, the long-term prognostic durability remains uncertain. This limitation highlights the need for future studies with extended follow-up to assess the predictive value of the 3-month mRS outcomes. Future studies should explore longitudinal outcomes at 6 months or 1 year to assess prognostic durability. Fourth, laboratory parameters such as neutrophils and lymphocytes were not included in our study, which prevented us from calculating the NLR and SII indices. In future research, we aim to incorporate a broader range of laboratory parameters to enhance the prediction of stroke outcomes. Fifth, the study cohort consisted exclusively of Korean patients, and the generalizability of these findings to other populations remains uncertain. Multicenter studies across diverse populations are needed to confirm the external validity of these results. Finally, further mechanistic research is needed to elucidate the biological pathways underlying the association between elevated PHR and adverse AIS outcomes.

## 5 Conclusion

This study revealed a nonlinear association between the PHR and 3-month unfavorable outcomes in older patients with AIS. The prognostic value of the PHR was more pronounced in patients with hyperlipidemia, and hs-CRP partially mediated this association. These findings support the potential utility of the PHR as a simple and accessible biomarker for outcome prediction in AIS patients.

## Data Availability

The datasets presented in this study can be found in online repositories. The names of the repository/repositories and accession number(s) can be found at: The dataset is publicly available through PLoS ONE and can be accessed at https://journals.plos.org/plosone/article?id=10.1371/journal.pone.0228738#sec019.
