# Supplementary material for: Platelet-to-hemoglobin ratio and stroke prognosis in older adults: a nonlinear and inflammation-mediated association
Source: Front Med (Lausanne). 2025 Sep 30;12:1643860. doi: 10.3389/fmed.2025.1643860 (PMC12518330; doi:10.3389/fmed.2025.1643860)
Supplement: Supplementary file 1 [file Data_Sheet_1.docx]

Table S1 Data dictionary and measurement methods for key variables

| **Variables** | **Meaning** | **DataCollection** | **Units/Encoding** |
| --- | --- | --- | --- |
| Age | Age | Electronic medical records | Years |
| Gender | Gender | Electronic medical records | 1=Male, 2=Female |
| Laboratory Parameters | WBC, HGB, HCT, FIB, PLT, MCV, TG, TC, LDL-C, BUN, Scr, ALT, FBG, and hs-CRP | Electronic medical records | Units vary depending on the lab test |
| Height and Weight (Stand) | Height and weight of the participant | Automatic scale (GL-150 model) | cm, kg |
| Height and Weight (not Stand) | Height and weight of the participant | Bedside scale for weight, tape measure for height | cm, kg |
| Smoking Status | Whether the participant has smoked in the past 6 months | Electronic medical records | 0=No, 1=Yes |
| Medical History | Hypertension, diabetes, previous stroke/TIA, coronary heart disease (CHD), hyperlipidemia, atrial fibrillation | Electronic medical records | 0=No, 1=Yes |
| Stroke Etiology | TOAST classification: 1=LAA, 2=SVO, 3=CE, 4=Other determined, 5=Undetermined | Electronic medical records | 1=LAA, 2=SVO, 3=CE, 4=Other, 5=Undetermined |
| mRS Score | Modified Rankin Scale score, assessing functional recovery | Electronic medical records, outpatient visits, structured telephone | 0-6 points |
| NIHSS Score | National Institutes of Health Stroke Scale score, assessing neurological severity | Electronic medical records, outpatient visits, structured telephone | 0-42 points |

DM, diabetes mellitus; TIA, transient ischemic attack; CHD, coronary heart disease; LAA, large-artery atherosclerosis; SVO, small vessel occlusion; CE, cardioembolism; mRS, modified Rankin scale; NIHSS, national institutes of health stroke scale; WBC, white blood cell; HGB, hemoglobin; HCT, hematocrit; FIB, fibrinogen; PLT, platelet; MCV, mean corpuscular volume; TG, triglyceride; TC, total cholesterol; HDL-C, high-density lipoprotein cholesterol; LDL-C, low-density lipoprotein cholesterol; BUN, blood urea nitrogen; Scr, serum creatinine; ALT, alanine aminotransferase; FBG, fasting blood glucose; hs-CRP, high-sensitivity C-reactive protein.

| Table S2 Assessment of collinearity among independent variables in the final regression model. | | | | |
| --- | --- | --- | --- | --- |
| **Variables** | **GVIF** | **Df** | **Adjusted GVIF** | **Collinearity** |
| Age | 1.13 | 2 | 1.031 | No |
| Gender | 1.525 | 1 | 1.235 | No |
| BMI | 1.12 | 1 | 1.058 | No |
| Smoking | 1.454 | 1 | 1.206 | No |
| Hypertension | 1.105 | 1 | 1.051 | No |
| DM | 1.078 | 1 | 1.037 | No |
| ALT | 1.049 | 1 | 1.024 | No |
| BUN | 1.979 | 1 | 1.407 | No |
| LDL-C | 1.172 | 1 | 1.083 | No |
| Previous stroke/TlA | 1.025 | 1 | 1.013 | No |
| Hyperlipidemia | 1.114 | 1 | 1.055 | No |
| Atrial fibrillation | 1.151 | 1 | 1.073 | No |
| CHD | 1.066 | 1 | 1.032 | No |
| NIHSS score at admission | 1.185 | 2 | 1.043 | No |
| FBG | 1.098 | 1 | 1.048 | No |
| Scr | 1.994 | 1 | 1.412 | No |
| WBC | 1.089 | 1 | 1.044 | No |
| BMI, body mass index; DM, diabetes mellitus; ALT, alanine aminotransferase; BUN, blood urea nitrogen; LDL-C, low-density lipoprotein cholesterol; TIA, transient ischemic attack; CHD, coronary heart disease; NIHSS, national institutes of health stroke scale; FBG, fasting blood glucose; Scr, serum creatinine; WBC, white blood cell. | | | | |

| Table S3 Baseline characteristics between participants favorable outcomes and unfavorable outcomes. | | | | |
| --- | --- | --- | --- | --- |
|  | **Total** | **Favorable outcomes** | **Unfavorable outcomes** | **P** |
| Participants | 1470 | 1008 | 462 |  |
| Gender |  |  |  | < 0.001 |
| Male | 854 (58.10) | 626 (62.1) | 228 (49.35) |  |
| Female | 616 (41.90) | 382 (37.9) | 234 (50.65) |  |
| Age (years) |  |  |  | < 0.001 |
| 60 to < 70 | 505 (34.35) | 396 (39.29) | 109 (23.59) |  |
| 70 to < 80 | 670 (45.58) | 461 (45.73) | 209 (45.24) |  |
| ≥ 80 | 295 (20.07) | 151 (14.98) | 144 (31.17) |  |
| BMI (kg/m2) |  |  |  | 0.002 |
| <25 | 1080 (73.47) | 721 (71.53) | 359 (77.71) |  |
| 25-29.9 | 358 (24.35) | 270 (26.79) | 88 (19.05) |  |
| >30 | 32 (2.18) | 17 (1.69) | 15 (3.25) |  |
| Smoking, n (%) |  |  |  | < 0.001 |
| No | 957 (65.10) | 621 (61.61) | 336 (72.73) |  |
| Yes | 513 (34.90) | 387 (38.39) | 126 (27.27) |  |
| Hypertension, n (%) |  |  |  | 0.031 |
| No | 464 (31.56) | 336 (33.33) | 128 (27.71) |  |
| Yes | 1006 (68.44) | 672 (66.67) | 334 (72.29) |  |
| DM, n (%) |  |  |  | 0.028 |
| No | 966 (65.71) | 681 (67.56) | 285 (61.69) |  |
| Yes | 504 (34.29) | 327 (32.44) | 177 (38.31) |  |
| Previous stroke/TIA, n (%) |  |  |  | < 0.001 |
| No | 1125 (76.53) | 804 (79.76) | 321 (69.48) |  |
| Yes | 345 (23.47) | 204 (20.24) | 141 (30.52) |  |
| CHD, n (%) |  |  |  | 0.478 |
| No | 1275 (86.73) | 870 (86.31) | 405 (87.66) |  |
| Yes | 195 (13.27) | 138 (13.69) | 57 (12.34) |  |
| Hyperlipidemia, n (%) |  |  |  | 0.125 |
| No | 948 (64.49) | 637 (63.19) | 311 (67.32) |  |
| Yes | 522 (35.51) | 371 (36.81) | 151 (32.68) |  |
| Atrial fibrillation, n (%) |  |  |  | < 0.001 |
| No | 1099 (74.76) | 791 (78.47) | 308 (66.67) |  |
| Yes | 371 (25.24) | 217 (21.53) | 154 (33.33) |  |
| Stroke etiology, n (%) |  |  |  | < 0.001 |
| LAA | 477 (32.45) | 339 (33.63) | 138 (29.87) |  |
| SVO | 288 (19.59) | 228 (22.62) | 60 (12.99) |  |
| CE | 427 (29.05) | 266 (26.39) | 161 (34.85) |  |
| Other determined | 98 ( 6.67) | 49 (4.86) | 49 (10.61) |  |
| Undetermined | 180 (12.24) | 126 (12.5) | 54 (11.69) |  |
| mRS at admission, n (%) |  |  |  | < 0.001 |
| 0 | 1058 (72.02) | 783 (77.68) | 275 (59.65) |  |
| 1 | 130 ( 8.85) | 95 (9.42) | 35 (7.59) |  |
| 2 | 90 ( 6.13) | 60 (5.95) | 30 (6.51) |  |
| 3 | 80 ( 5.45) | 44 (4.37) | 36 (7.81) |  |
| 4 | 61 ( 4.15) | 14 (1.39) | 47 (10.2) |  |
| 5 | 50 ( 3.40) | 12 (1.19) | 38 (8.24) |  |
| mRS at admission, n (%) |  |  |  | < 0.001 |
| ≤ 2 | 220 (53.53) | 155 (68.89) | 65 (34.95) |  |
| ≥ 3 | 191 (46.47) | 70 (31.11) | 121 (65.05) |  |
| NIHSS score at admission, n (%) |  |  |  | < 0.001 |
| ≤ 5 | 864 (58.78) | 745 (73.91) | 119 (25.76) |  |
| 5 to ≤ 13 | 469 (31.90) | 232 (23.02) | 237 (51.3) |  |
| > 13 | 137 ( 9.32) | 31 (3.08) | 106 (22.94) |  |
| WBC (10^9/L) | 8.04 ± 2.88 | 7.86 ± 2.64 | 8.43 ± 3.31 | < 0.001 |
| HGB (g/L) | 132.5 ± 19.6 | 134.7 ± 18.2 | 127.8 ± 21.7 | < 0.001 |
| HCT (%) | 39.49 ± 5.52 | 40.08 ± 5.14 | 38.21 ± 6.08 | < 0.001 |
| FIB (mg/L) | 332.93 ± 92.03 | 325.42 ± 86.90 | 349.30 ± 100.49 | < 0.001 |
| PLT (×10⁹/L) | 219.04 ± 69.99 | 218.96 ± 64.58 | 219.21 ± 80.61 | 0.949 |
| MCV | 93.34 ± 5.03 | 93.47 ± 4.65 | 93.07 ± 5.78 | 0.161 |
| TG (mg/dl) | 101.68 ± 55.26 | 105.42 ± 56.08 | 93.54 ± 52.58 | < 0.001 |
| TC (mg/dl) | 175.59 ± 42.77 | 177.19 ± 41.26 | 172.10 ± 45.75 | 0.034 |
| HDL-C (mg/dl) | 43.98 ± 16.67 | 44.40 ± 16.13 | 43.06 ± 17.79 | 0.152 |
| LDL-C (mg/dl) | 101.68 ± 41.35 | 102.93 ± 39.40 | 98.97 ± 45.25 | 0.088 |
| BUN (mg/dl) | 18.43 ± 9.34 | 18.09 ± 8.68 | 19.16 ± 10.60 | 0.04 |
| Scr (mg/dl) | 0.90  (0.74, 1.11) | 0.91  (0.76, 1.10) | 0.87  (0.70, 1.12) | 0.109 |
| ALT (U/L) | 18.00  (13.00, 25.00) | 18.00  (14.00, 26.00) | 17.00  (11.00, 25.00) | < 0.001 |
| FBG (mg/dl) | 99.17 ± 45.86 | 97.98 ± 39.52 | 101.76 ± 57.27 | 0.142 |
| hs-CRP (mg/L) | 0.13  (0.04, 0.48) | 0.10  (0.03, 0.32) | 0.26  (0.07, 1.82) | < 0.001 |
| PHR | 1.69 ± 0.66 | 1.66 ± 0.57 | 1.78 ± 0.81 | 0.001 |
| Note: Variables are presented as the means ± SDs, medians (IQRs) or n (%).  BMI, body mass index; DM, diabetes mellitus; TIA, transient ischemic attack; CHD, coronary heart disease; LAA, large-artery atherosclerosis; SVO, small vessel occlusion; CE, cardioembolism; mRS, modified Rankin scale; NIHSS, national institutes of health stroke scale; WBC, white blood cell; HGB, hemoglobin; HCT, hematocrit; FIB, fibrinogen; PLT, platelet; MCV, mean corpuscular volume; TG, triglyceride; TC, total cholesterol; HDL-C, high-density lipoprotein cholesterol; LDL-C, low-density lipoprotein cholesterol; BUN, blood urea nitrogen; Scr, serum creatinine; ALT, alanine aminotransferase; FBG, fasting blood glucose; hs-CRP, high-sensitivity C-reactive protein; PHR, platelet-to-hemoglobin ratio. | | | | |

| Table S4 Baseline characteristics between participants favorable outcomes and unfavorable outcomes. | | |
| --- | --- | --- |
| **Variables** | **Odd ratios (95% CI)** | **P** |
| Gender |  |  |
| Male | 1 |  |
| Female | 1.682 (1.347, 2.101) | <0.001 |
| Age (years) |  |  |
| 60 to < 70 | 1 |  |
| 70 to < 80 | 1.647 (1.26, 2.153) | <0.001 |
| ≥ 80 | 3.465 (2.537, 4.731) | <0.001 |
| BMI (kg/m2) |  |  |
| <25 | 1 |  |
| 25-29.9 | 0.655 (0.499, 0.859) | 0.002 |
| >30 | 1.772 (0.875, 3.589) | 0.112 |
| Smoking |  |  |
| No | 1 |  |
| Yes | 0.602 (0.473, 0.766) | <0.001 |
| Hypertension |  |  |
| No | 1 |  |
| Yes | 1.305 (1.024, 1.662) | 0.031 |
| DM |  |  |
| No | 1 |  |
| Yes | 1.293 (1.028, 1.627) | 0.028 |
| Previous stroke/TIA |  |  |
| No | 1 |  |
| Yes | 1.731 (1.347, 2.224) | <0.001 |
| CHD |  |  |
| No | 1 |  |
| Yes | 0.887 (0.638, 1.235) | 0.478 |
| Hyperlipidemia |  |  |
| No | 1 |  |
| Yes | 0.834 (0.661, 1.052) | 0.126 |
| Atrial fibrillation |  |  |
| No | 1 |  |
| Yes | 1.823 (1.427, 2.328) | <0.001 |
| Stroke etiology |  |  |
| LAA | 1 |  |
| SVO | 0.646 (0.457, 0.914) | 0.014 |
| CE | 1.487 (1.126, 1.964) | 0.005 |
| Other determined | 2.457 (1.578, 3.825) | <0.001 |
| Undetermined | 1.053 (0.723, 1.532) | 0.788 |
| mRS at admission |  |  |
| 0 | 1 |  |
| 1 | 1.049 (0.695, 1.583) | 0.820 |
| 2 | 1.424 (0.899, 2.254) | 0.132 |
| 3 | 2.33 (1.469, 3.695) | <0.001 |
| 4 | 9.559 (5.181, 17.634) | <0.001 |
| 5 | 9.016 (4.644, 17.504) | <0.001 |
| mRS at admission |  |  |
| ≤ 2 | 1 |  |
| ≥ 3 | 4.122 (2.728, 6.229) | <0.001 |
| NIHSS score at admission |  |  |
| ≤ 5 | 1 |  |
| 5 to ≤ 13 | 6.395 (4.907, 8.336) | <0.001 |
| > 13 | 21.407 (13.725, 33.389) | <0.001 |
| Laboratory parameters |  |  |
| WBC (10^9/L) | 1.07 (1.031, 1.111) | <0.001 |
| HGB (g/dL) | 0.838 (0.792, 0.887) | <0.001 |
| HCT (%) | 0.94 (0.922, 0.96) | <0.001 |
| FIB (mg/L) | 1.003 (1.002, 1.004) | <0.001 |
| PLT (×10⁹/L) | 1 (0.998, 1.002) | 0.949 |
| MCV | 0.984 (0.963, 1.006) | 0.161 |
| TG (mg/dl) | 0.996 (0.994, 0.998) | <0.001 |
| TC (mg/dl) | 0.997 (0.995, 1) | 0.035 |
| HDL-C (mg/dl) | 0.995 (0.989, 1.002) | 0.152 |
| LDL-C (mg/dl) | 0.998 (0.995, 1) | 0.088 |
| BUN (mg/dl) | 1.012 (1, 1.023) | 0.042 |
| Scr (mg/dl) | 1.031 (0.93, 1.144) | 0.56 |
| ALT (U/L) | 0.992 (0.984, 1) | 0.057 |
| FBG (mg/dl) | 1.002 (0.999, 1.004) | 0.143 |
| hs-CRP (mg/L) | 1.136 (1.092, 1.182) | <0.001 |

Note: Variables are presented as the means ± SDs, medians (IQRs) or n (%).

BMI, body mass index; DM, diabetes mellitus; TIA, transient ischemic attack; CHD, coronary heart disease; LAA, large-artery atherosclerosis; SVO, small vessel occlusion; CE, cardioembolism; mRS, modified Rankin scale; NIHSS, national institutes of health stroke scale; WBC, white blood cell; HGB, hemoglobin; HCT, hematocrit; FIB, fibrinogen; PLT, platelet; MCV, mean corpuscular volume; TG, triglyceride; TC, total cholesterol; HDL-C, high-density lipoprotein cholesterol; LDL-C, low-density lipoprotein cholesterol; BUN, blood urea nitrogen; Scr, serum creatinine; ALT, alanine aminotransferase; FBG, fasting blood glucose; hs-CRP, high-sensitivity C-reactive protein.

| Table S5 ROC curve analysis of thresholds for PHR, HGB, and PLT in predicting unfavorable outcomes. | | | | | | |
| --- | --- | --- | --- | --- | --- | --- |
| **Variables** | **Threshold** | **Sensitivity (95% CI)** | **Specificity**  **(95% CI)** | **PPV**  **(95% CI)** | **NPV**  **(95% CI)** | **Brier score** |
| **PHR** | 1.869 | 0.558  (0.533, 0.584) | 0.586  (0.561, 0.611) | 0.382  (0.357, 0.407) | 0.743  (0.721, 0.766) | 0.209 |
| **HGB (g/L)** | 132.5 | 0.351  (0.327, 0.375) | 0.748  (0.725, 0.77) | 0.389  (0.364, 0.414) | 0.715  (0.692, 0.738) | 0.214 |
| **PLT (×10⁹/L)** | 255.5 | 0.258  (0.236, 0.281) | 0.777  (0.755, 0.797) | 0.346  (0.322,0.37) | 0.695  (0.672, 0.719) | 0.216 |

HGB, hemoglobin; PLT, platelet; PHR, platelet-to-hemoglobin ratio.

| Table S6 Mediation analysis of the effect of PHR on 3-month unfavorable outcomes through hs-CRP. | | |
| --- | --- | --- |
| **Effect Type** | **OR (95% CI )** | **P** |
| **Model-based estimates** |  |  |
| Average Causal Mediation Effect | 0.01 (0.004, 0.017) | 0.002 |
| Average Direct Effect | 0.028 (0.002, 0.053) | 0.042 |
| Total Effect | 0.038 (0.009, 0.062) | 0.016 |
| Proportion Mediated (%) | 25.99 (6.67, 98.95) | 0.018 |
| **Bootstrap estimates (5,000 resamples)** |  |  |
| Average Causal Mediation Effect | 0.01 (0.004, 0.017) | 0.006 |
| Average Direct Effect | 0.028 (0.001, 0.055) | 0.044 |
| Total Effect | 0.038 (0.004, 0.061) | 0.032 |
| Proportion Mediated (%) | 26.59 (3.96, 123.86) | 0.038 |

Adjusted for age, gender, BMI, WBC, ALT, BUN, Scr, LDL-C, FBG,smoking, previous stroke/TIA, hypertension, DM, hyperlipidemia, AF,CHD, stroke etiology, and NIHSS score at admission.
