# Supplementary figures and images for: Platelet-to-hemoglobin ratio and stroke prognosis in older adults: a nonlinear and inflammation-mediated association
Source: Front Med (Lausanne). 2025 Sep 30;12:1643860. doi: 10.3389/fmed.2025.1643860 (PMC12518330; doi:10.3389/fmed.2025.1643860)

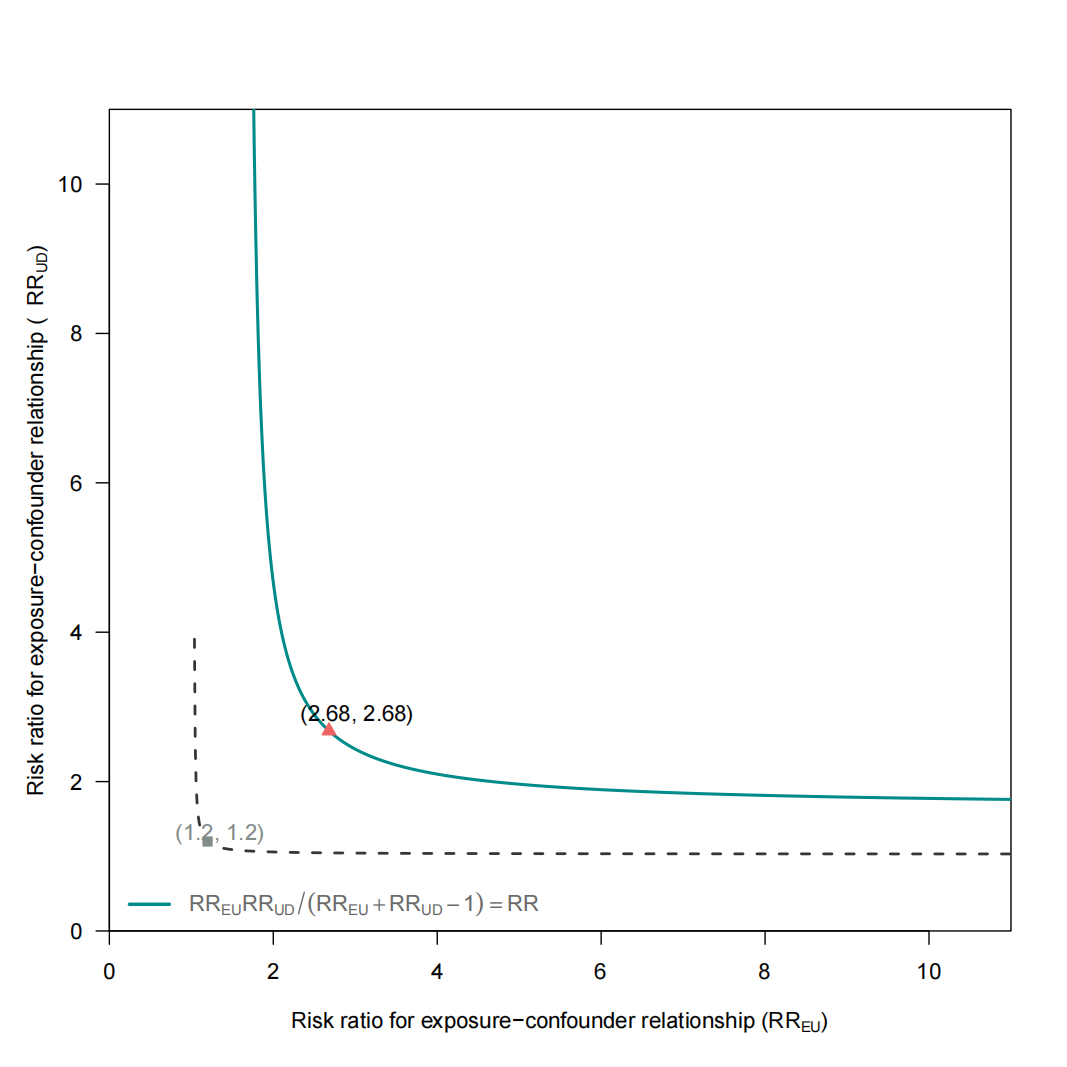

Supplement: Supplementary file 2 [file Image_1.tif]
